# Human umbilical cord mesenchymal stem cells therapy alleviates kidney injury and podocyte apoptosis in *Col4a5* knockout male mice

**DOI:** 10.1002/ctm2.70506

**Published:** 2025-10-20

**Authors:** Di Lu, Zhitao Ye, Liujing Xu, Shumin Zhou, Guanyu Li, Jiayi Zhang, Yi Liu, Yue Li, Qizhou Lian, Zheng Shen, Jiao Lin, Qi Wang, Xia Gao

**Affiliations:** ^1^ Department of Nephrology Guangzhou Women and Children's Medical Center Guangzhou Medical University Guangzhou China; ^2^ Maoming Maternal and Child Health Hospital Maoming China; ^3^ Biomedical Pioneering Innovation Center (BIOPIC) and School of Life Sciences Peking University Beijing China; ^4^ Beihao Stem Cell and Regenerative Medicine Research Institute Co. Ltd. Guangzhou China

1

Dear Editor,

The X‐linked form of Alport syndrome (AS), resulting from COL4A5 defects, comprises 80%–85% of AS cases and exhibits particularly aggressive progression in male patients, with most progressing to renal failure during early adulthood.^1^, ^2^ Current treatment options remain limited to nonspecific interventions and supportive care. While mesenchymal stem cells from human umbilical cord (hUC‐MSCs) demonstrate benefits in various kidney diseases, their application in genetic disorders such as AS remains unexplored.^3^, ^4^, ^5^, ^6^ This study demonstrated that hUC‐MSCs treatment extended survival and ameliorated renal injury and podocyte apoptosis in male *Col4a5* knockout (KO) mice, highlighting its potential as a therapeutic strategy for AS.

We generated *Col4a5* KO mice on the C57/BL6JGpt background through CRISPR/Cas9 technology (Figures 1A and S1A,B). The renal function and pathology of these mice were monitored at different time points (Figure 1B). Compared to wild‐type (WT) littermates, KO mice exhibited spontaneous mortality between 127 and 211 days of age, accompanied by retarded body weight gain (Figure 1C,D). Changes in kidney function markers emerged at 7 weeks of age with increased ACR and Scr levels (Figure 1G,H), followed by elevated Blood Urea Nitrogen (BUN) at 14 weeks (Figure 1I) and higher cystatin C at 21 weeks (Figure 1J). Histologically, while renal morphology appeared largely normal in KO mice at 7 weeks, focal glomerulosclerosis emerged at 14 weeks, followed by tubular injury and mild interstitial fibrosis at 21 weeks. By 28 weeks, extensive glomerulosclerosis, inflammatory cell infiltration and interstitial fibrosis were observed (Figure 1K‒N). Gross examination of kidneys at 28 weeks revealed renal atrophy and reduced blood perfusion in KO mice.

**FIGURE 1:**
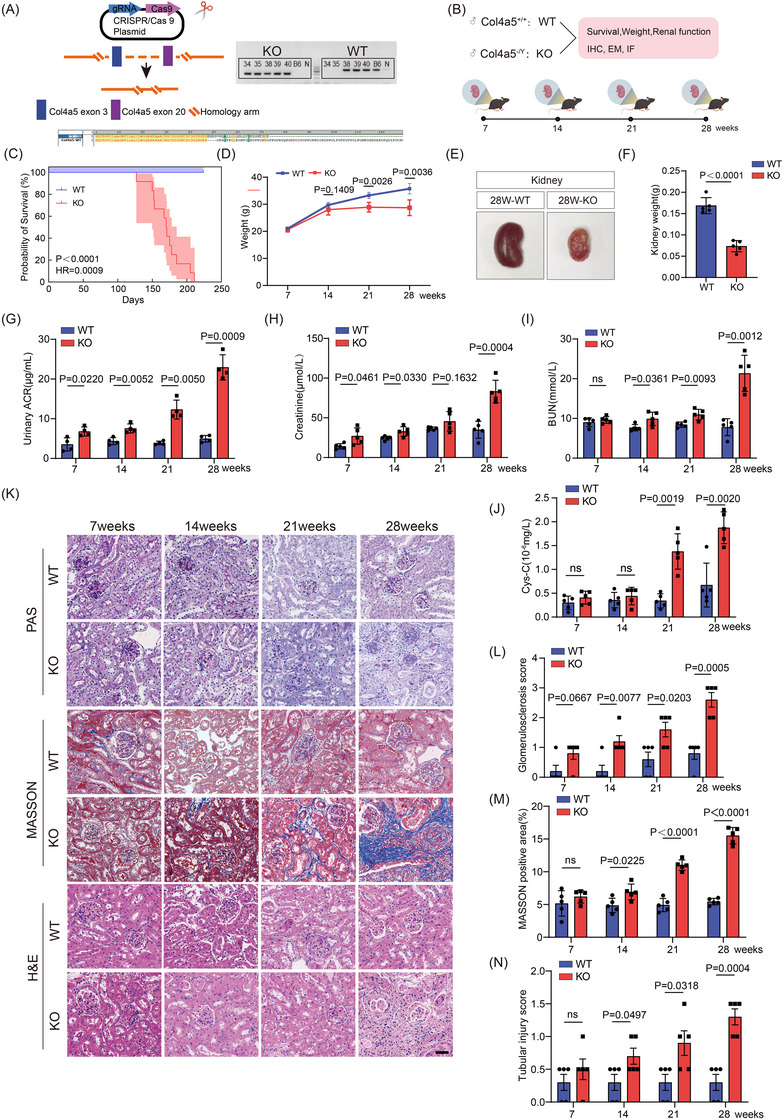
*Col4a5* gene knockout (KO) and phenotypic verification in mice. (A) CRISPR/Cas9 targeting strategy of *COL4A5* gene (left). Genotyping validation (right). Amino acid sequence alignment between wild‐type (WT) and KO (bottom). (B) Experimental design. (C) Survival curves of WT and *Col4a5* KO mice (*n* = 12). (D) Body weight of WT and *Col4a5* KO mice at different ages. (E and F) Kidney appearance and kidney weight of WT and *Col4a5* KO mice at 28 weeks of age (*n* = 5). (G‒J) Urine protein/creatinine ratio (ACR) (G), serum creatinine (Scr) (H), serum urea nitrogen (I) and serum cystatin C (J) of WT and *Col4a5* KO mice at 7, 14, 21 and 28 weeks of age (*n* = 5). (K‒N) PAS, MTC and HE staining (K), glomerular sclerosis score (L), Masson staining positive area (M) and tubular damage score (N) of WT and *Col4a5* KO mice (*n* = 5). Scale bar: 50 µm. Two‐tailed *T*‐test was used for samples and *p*<.05 was statistically significant. PAS, periodic acid‐schiff stain; MTC, masson trichrome staining; HE, hematoxylin‐eosin staining.

The multipotency of isolated hUC‐MSCs was verified by examining their stemness markers and trilineage differentiation potential (Figure S1C‒E). After intravenous injection of fluorescently labelled hUC‐MSCs (2 × 10^5^ cells/mouse, Passage3) at weeks 9 and 11 (Figure 2A), cells exhibited optimal kidney homing at 24 h post‐injection and remained detectable for up to 120 h (Figure S2A‒C). hUC‐MSCs treatment significantly improved survival rates in Col4a5 KO mice (Figure 2B), ameliorated growth retardation (Figure 2C) and improved urine output (Figure 2D,E). The treatment also reduced the proportion of mice with severe proteinuria and 3+ haematuria in late‐stage disease (Figure 2G,H). In treated KO mice, we observed reduced ACR at 21 weeks (Figure 2I), along with delayed elevation of Scr (Figure 2J), BUN (Figure 2K) and cystatin C (Figure 2L). Gross examination of kidneys at 28 weeks revealed that hUC‐MSCs treatment attenuated renal atrophy and increased renal blood flow in KO mice (Figure 2F). Histopathological analysis demonstrated that hUC‐MSCs treatment mitigated glomerulosclerosis (Figure 3A‒D), reduced podocyte detachment (Figure 3E,F) and partially restored glomerular basement membrane structure (Figure 3G,H).

**FIGURE 2:**
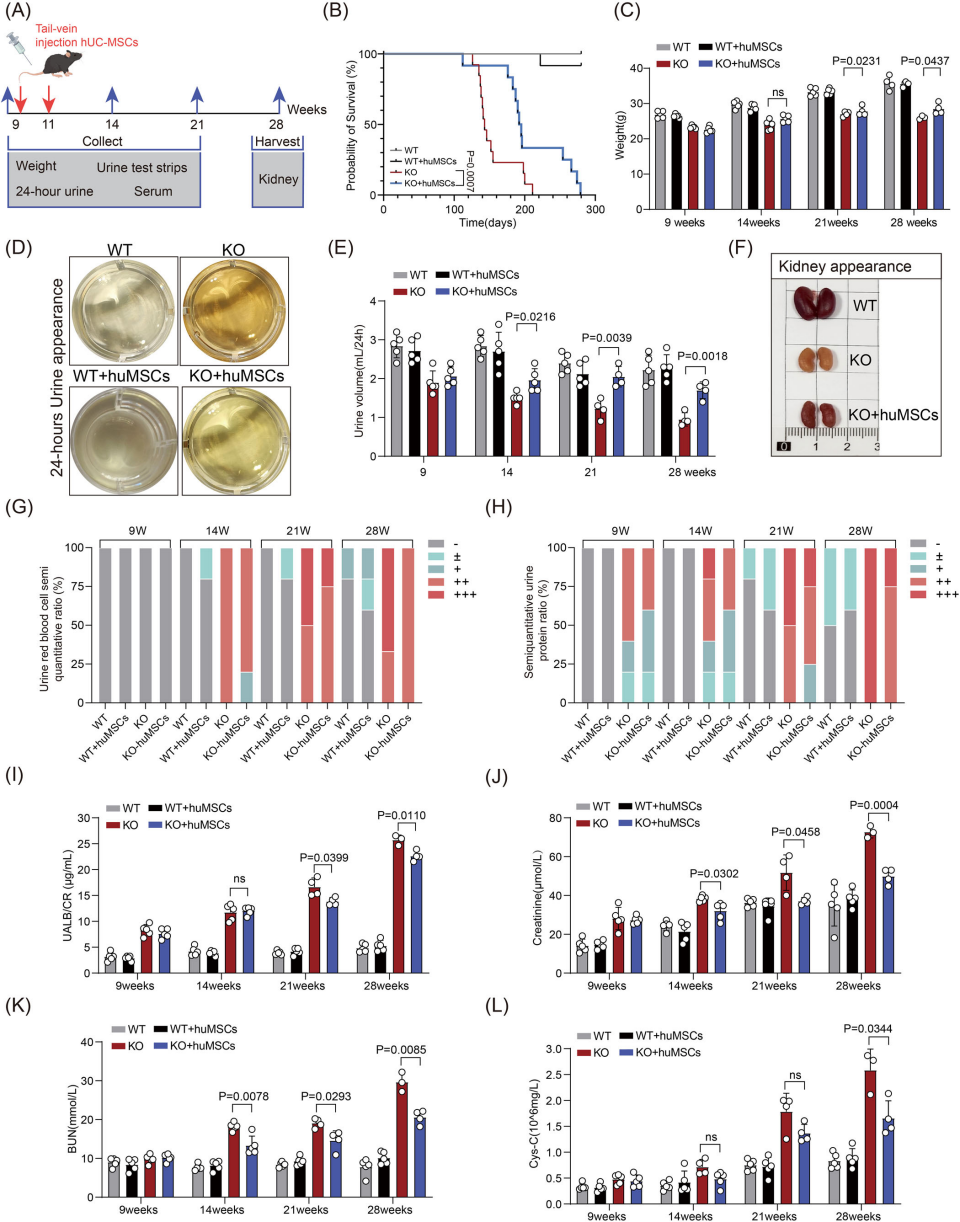
Mesenchymal stem cells from human umbilical cord (hUC‐MSCs) treatment alleviates renal function in *Col4a5* knockout (KO) mice. (A) Experimental design: hUC‐MSCs were labelled with 1,1′‐dioctadecyl‐3,3,3′,3′‐tetramethylindotricarbocyanine iodide (DiR) and then injected into wild‐type (WT) and KO mice at 9 and 11 days, and kidneys were harvested at 28 weeks. (B) Survival analysis of hUC‐MSCs‐treated *Col4a5* KO mice (*n* = 12). (C‒H) Administration of hUC‐MSCs at 9 and 11 weeks of age showing body weight (C), gross appearance of urine (D), 24‐h urine volume (E), gross morphology of kidneys (F), semi‐quantitative analysis of haematuria (G) and proteinuria (H) in *Col4a5*‐deficient mice at 9, 14, 21 and 28 weeks of age (*n* = 5). Semi‐quantitative detection of urinary red blood cells: ‒, negative; +, 25 cell/µL; ++, 80 cell/µL; +++, ≥200 cell/µL. Semi‐quantitative urine protein test: ‒, negative; ±, .15 g/L; +, .3 g/L; ++, 1.0 g/L; +++, 3.0 g/L. Data are expressed as mean ± standard deviation (SD). (I‒L) Quantitative analysis of urinary albumin‐to‐creatinine ratio (UACR) (I), serum creatinine (Scr) (J), blood urea nitrogen (K) and serum cystatin C (L) after hUC‐MSCs treatment (*n* = 5). Two‐way analysis of variance (ANOVA) and Tukey's post hoc test were used for the samples. *p* < .05 was statistically significant.

**FIGURE 3:**
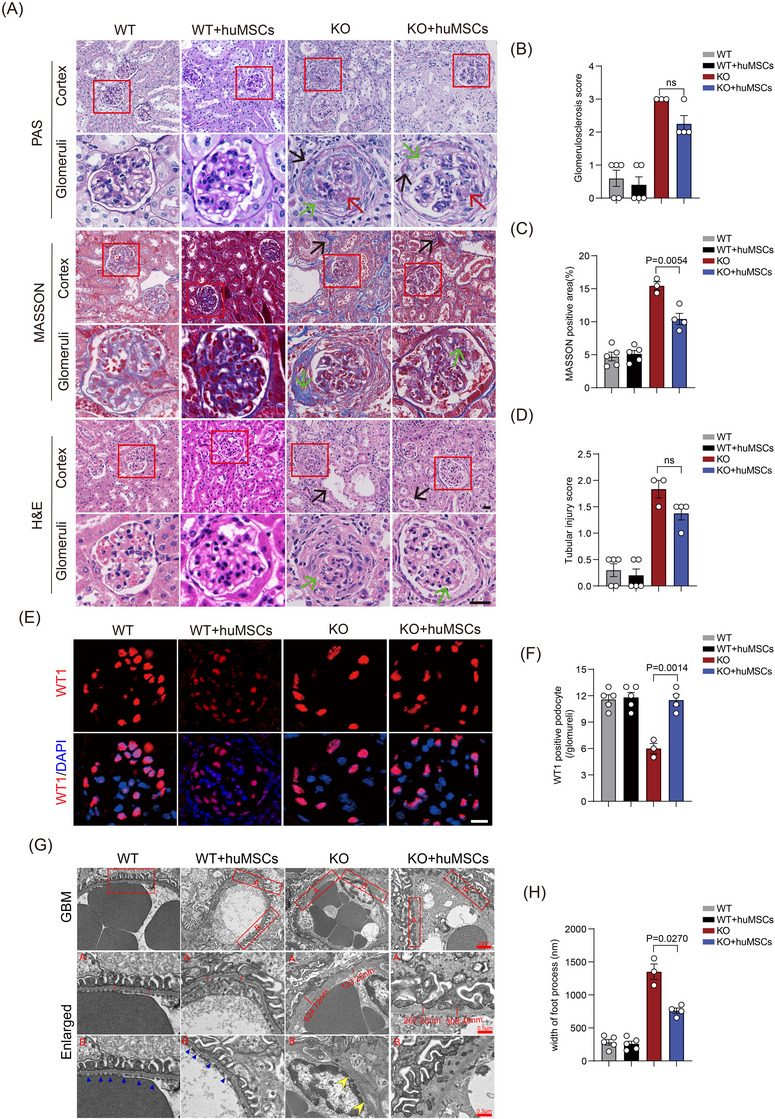
Mesenchymal stem cells from human umbilical cord (hUC‐MSCs) treatment improves renal pathology in *Col4a5* knockout mice. (A‒D) Representative images and quantification of PAS (green arrows: Bowman's capsule thickening; red arrows: glomerulosclerosis; black arrows: detached podocytes), Masson's trichrome (black arrows: interstitial fibrosis; green arrows: glomerular fibrosis) and HE staining (green arrows: irregular GBM thickness; black arrows: loss of tubular brush border) of kidneys from *Col4a5* knockout mice at 28 weeks after hUC‐MSCs treatment. Scale bar: 20 µm. (E) Immunofluorescence of WT‐1 in glomeruli, as well quantification. Scale bar: 20 µm. (F) The structure of glomerular basement membrane under electron microscope. Scale bar: 2 µm (enlarged .5 µm). Data are expressed as mean ± standard deviation (SD). One‐way analysis of variance (ANOVA) and Tukey's post hoc test were used for the samples. *p* < .05 was statistically significant. Five mice/group. GBM, glomerular basement membrane. HE, Hematoxylin‐Eosin staining.

Integrins, as transmembrane receptors, mediate cell‒matrix adhesion by binding to extracellular matrix components such as type IV collagen.^7^ Podocytes express multiple integrins (α3β1, α1β1, α2β1 and αvβ3) that maintain stable adhesion with the Glomerular basement membrane (GBM) type IV collagen network and regulate cytoskeletal structure and cell survival through the FAK signalling pathway.^8^ In this process, FAK serves as a crucial downstream effector, and its autophosphorylation is a critical event in integrin signal transduction.^9^ To explore protective mechanisms of hUC‐MSCs, we established an in vitro model using podocytes differentiated from AS patient‐derived Pluripotent Stem Cells (iPSCs) (Figures 4A and S3A‒C). Our studies found that the deletion of type IV collagen α5 chain (COL4A5) led to altered podocyte morphology from spindle to round shape (manifested as increased proportion of type C and type D morphology) and enhanced cell apoptosis (Figure 4B‒D,G,H). *COL4A5* deficiency resulted in decreased phosphorylation of downstream effectors FAK and AKT (Figure 4L,M), downregulation of anti‐apoptotic protein BCL‐2 (Figure 4K) and increased expression of pro‐apoptotic molecule caspase3 (Figure 4I,J). Previous studies have shown that hUC‐MSCs can inhibit cell apoptosis by secreting Insulin‐like Growth Factor (IGF‐1) to activate the PI3K/Akt/FOXO3 signalling pathway.^10^ Co‐culture with hUC‐MSCs resulted in significantly elevated IGF‐1 levels in the conditioned medium and increased IGF‐1 mRNA expression (Figure 4E,F). This co‐culture system improved cell morphology, attenuated apoptosis and restored AKT phosphorylation in *COL4A5*‐deficient podocytes, while FAK phosphorylation remained impaired. These results suggest that hUC‐MSCs‐secreted IGF‐1 may partially compensate for podocyte apoptosis caused by impaired integrin‒FAK signalling.

**FIGURE 4:**
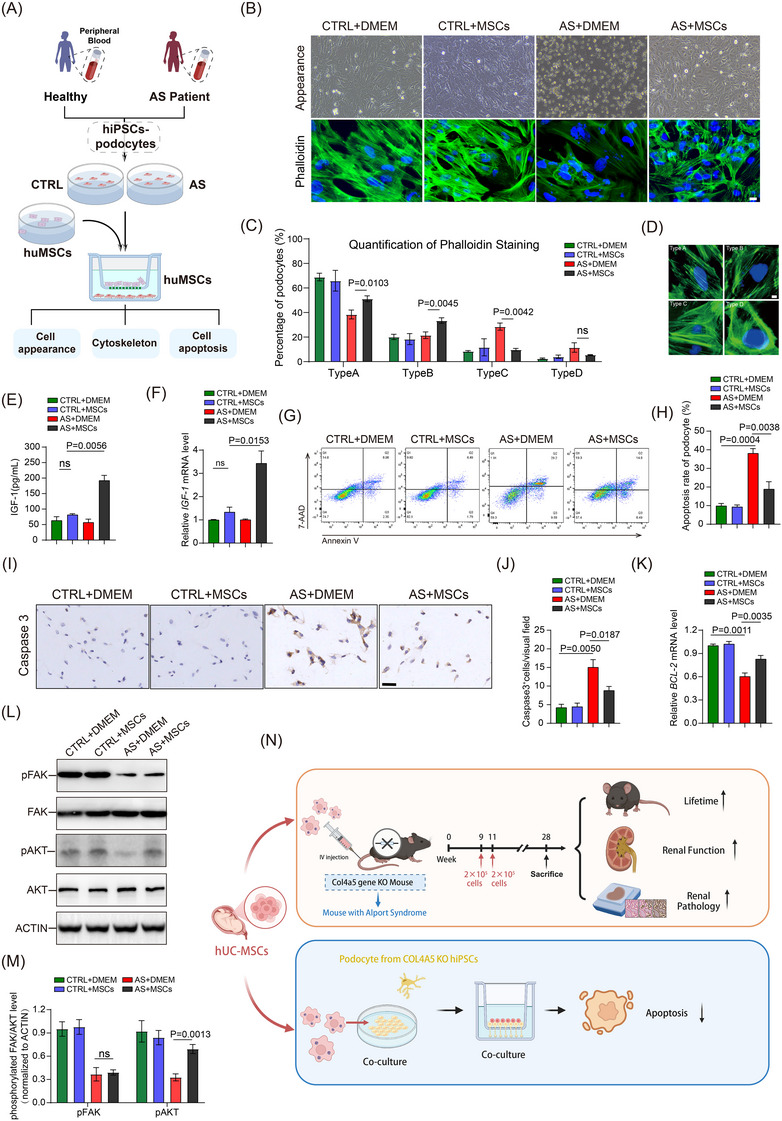
Co‐culture of mesenchymal stem cells from human umbilical cord (hUC‐MSCs) with podocytes from patients with Alport syndrome reduced cell apoptosis. (A) Experimental design diagram: iPSC‐derived podocytes from healthy donors and Alport syndrome patients were co‐cultured with DMEM (CTRL) or hUC‐MSCs for 48 h. (B) Representative phase‐contrast images and phalloidin staining of hUC‐MSCs co‐cultured with Alport syndrome‐derived differentiated podocytes. Scale bar: 100 µm. (C and D) Quantitative analysis and representative images of different phalloidin staining patterns in podocytes (*n* = 3). Scale bar 10 µm. (E) The IGF‐1 level in cultural supernatant of different treatment groups was detected by ELISA assay (*n* = 3). (F) Relative *IGF‐1* mRNA expression levels in different treatment groups were analysed by real‐time quantitative PCR (RT‐qPCR) (*n* = 3). (G and H) Flow cytometric analysis of podocyte apoptosis and quantification of apoptotic percentage among different groups. (I and J) Representative immunohistochemical images and quantitative analysis of caspase3‐positive cells across treatment groups (n = 3). Scale bar: 50 µm. (K) RT‐qPCR analysis of anti‐apoptotic gene *BCL‐2* expression (*n* = 3). (L and M) Representative Western blot images and densitometric analysis of total and phosphorylated FAK and AKT levels in different treatment groups. Data were normalised to ACTIN. (N) Visual overview of main research outcomes. Data are expressed as mean ± standard deviation (SD). One‐way analysis of variance (ANOVA) and Tukey's post hoc test were used for the samples. *p* < .05 was statistically significant. IGF‐1, insulin‐like growth factor; DMEM, dulbecco's modified eagle medium; iPSC, pluripotent stem cells; ELISA, enzyme‐linked immunosorbent assay

In conclusion, our study demonstrates that hUC‐MSCs effectively ameliorate proteinuria in male *Col4a5*‐deficient mice (Figure 4N). We revealed that IGF‐1 secreted by hUC‐MSCs reduces podocyte apoptosis caused by *COL4A5* deficiency through enhanced Akt phosphorylation. Although we were unable to fully evaluate the efficacy through comparison or combination with conventional ACEI therapy, our findings suggest promising applications of stem cells in treating paediatric genetic disorders. These results establish a foundation for future clinical trials and underscore the promise of hUC‐MSCs as a novel treatment approach for AS.

## AUTHOR CONTRIBUTIONS

Xia Gao and Qi Wang conceived and supervised the research. Di Lu, Zhitao Ye and Guanyu Li prepared the initial manuscript draft. Di Lu, Zhitao Ye, Liujing Xu, Guanyu Li and Shumin Zhou performed the experiments. Statistical evaluation was performed by Di Lu, Zhitao Ye, Jiayi Zhang and Yi Liu, with Qizhou Lian and Yue Li managing laboratory resources and technical aspects. All contributors have validated the final document.

## CONFLICT OF INTEREST STATEMENT

The authors declare they have no conflicts of interest.

## ETHICS STATEMENT

The Animal Ethics Committee of Guangdong Huawei Testing Co. Ltd. (2021‐HWT‐BG‐117) approved this study.

## Supporting information



Supporting Information

## Data Availability

Research materials and Supporting Information can be made available by contacting the senior corresponding author.
