# Supplementary material for: Human umbilical cord mesenchymal stem cells therapy alleviates kidney injury and podocyte apoptosis in Col4a5 knockout male mice
Source: Clin Transl Med. 2025 Oct 20;15(10):e70506. doi: 10.1002/ctm2.70506 (PMC12536887; doi:10.1002/ctm2.70506)
Supplement: Supplementary file 1 — Supporting Information [file CTM2-15-e70506-s001.docx]

**Supplementary Material**

| Catalog | | Pg No |
| --- | --- | --- |
| **Method** | | 2 |
| **Supplementary Figure** | |  |
|  | Figure S1 Validation of *Col4a5* knockout mouse model and characterization of isolated hUC-MSCs. | 7 |
|  | Figure S2 Distribution of hUC-MSCs in kidneys of WT and *Col4a5* knockout mice. | 8 |
|  | Figure S3 Identification of renal podocytes differentiated from human induced pluripotent stem cells (hiPSCs). | 9 |

**Method**

**Construction and Identification of *Col4a5* Knockout Mouse Model**

This study was approved by the Animal Ethics Committee of Guangzhou Women and Children's Medical Center. Using the MIT CRISPR Design tool, we designed gRNA sequences targeting the Col4a5 gene and selected two high-scoring gRNA recognition sequences in the intron 2-3 and intron 20-21 regions, which were subsequently cloned into the pX330 vector. Following PAGE purification and annealing of the gRNA sequences into double-stranded DNA, the recombinant plasmid was transformed into DH5α competent cells, with positive clones identified through kanamycin resistance selection and sequencing. The validated gRNA expression vector was linearized and purified for in vitro transcription using the MEGAshortscript Kit to generate gRNA, while Cas9 mRNA was transcribed from the pX330-gRNA plasmid template using the HiScribe T7 kit. The purified transcription products (Cas9 mRNAs at 100 ng/μl and Col4a5-gRNA at 50 ng/μl) were co-injected into C57BL/6Gpt fertilized eggs, resulting in knockout mice with a deletion of approximately 55.6kb spanning exons 3-20, as confirmed by genotyping and sequencing analysis. The Col4a5 knockout mice, provided by Guangdong Ji Cui Yao Kang, were maintained under controlled temperature and humidity conditions until seven weeks of age, with free access to food and water under a 12-hour light-dark cycle. The following are the primer sequences for col4a5 knockout mice and wild-type control mice in the same littermate:

WT Forward F5'-CATGCCAGGATTGTAGAATCAAAGG-3';

Reverse 5-'TCTGCCATCTCTAGTGCTACCTGGA-3';

KO Forward 5'-CTAGCTTACATGGTAAA CTACAGGTGC-3';

Reverse 5'-TAAGAGCCTGGAATGGTAATAATGG-3').

**Animal Groups and Treatment Protocol**

The experimental animals were classified into three groups, with each group comprising 5 mice: 1) The control group consists of WT individuals who do not get any intervention. 2) The disease group consists of KO individuals who receive saline. 3) The experimental group consists of KO individuals who receive human mesenchymal stem cells (huMSC). After the development of AS in the mouse model at 7 weeks old, a 2-week time was given for the mice to adjust to feeding themselves and drinking water. The environmental conditions were controlled to uphold a temperature range of 21-26°C, humidity levels between 50%-60%, and a 12-hour alternating cycle of light and darkness. Starting at nine weeks old, the mice were given hUC-MSC therapy twice, at nine and eleven weeks old, with a dosage of 2×10^5^ cells per mouse.

**Histological analysis**

The tissues were fixed in 4% paraformaldehyde for a minimum of 24 hours, then embedded in paraffin and sectioned for histological examination. Staining procedures followed the instructions provided by the manufacturer (Servicebio, Wuhan, China) for H&E, PAS, and Masson's trichrome staining. Image acquisition was carried out using Case Viewer software. Subsequently, ImageJ software was utilized for the quantitative analysis of collagen content following Masson's staining.

**Immunofluorescence staining**

The kidney tissues were fixed in 4% paraformaldehyde for 15 minutes and permeabilized with 0.2% Triton X-100 for an additional 15 minutes. Following this, immunostaining on glass slides was conducted using a primary antibody, succeeded by a fluorescently labeled secondary antibody. Nuclear counterstaining was accomplished with DAPI, and the samples were visualized using a Leica SP8 confocal microscope for image acquisition. Quantitative analysis of immunofluorescence was performed using ImageJ software.

**Transmission electron microscopy (TEM)**

Renal cortex tissue pieces measuring 1mm³ were immersed in electron microscopy fixative, dehydrated using a gradient, embedded, and then prepared for slicing at a thickness of 40-50 nm for electron microscopy. Following staining, the tissue sections were examined, and images were captured using a transmission electron microscope.

**Cell Culture**

**hUC-MSCs identification**

The hUC-MSCs used in this study were supplied by the GMP Laboratory of Coronado Biosciences. These cells were derived from umbilical cords obtained from healthy pregnant women who tested negative for HIV, syphilis antibodies, five hepatitis markers, TORCH panel, among others. Prior to cell collection, all participants provided informed consent. The cells were isolated, cultured, and their surface markers were assessed via flow cytometry. The analysis revealed high expression levels of CD73, CD90, and CD105, while showing no expression of CD11b, CD19, CD34, CD45, or HLA-DR. These findings confirmed the identity of the cells as mesenchymal stem cells.

**Human induced pluripotent stem cells (hiPSCs)**

Peripheral venous blood samples were collected from both healthy individuals and AS patients to isolate mononuclear cells. These cells were then differentiated into renal podocytes under various culture conditions using complete and conditional culture media. Following differentiation, the cells were seeded onto slides embedded in Matrigel, fixed with 4% paraformaldehyde, and characterized for podocyte markers including Podocin, WT-1, Synaptopodin, and stained with FITC-phalloidin to identify the hiPSCs.

***In Vivo* tracking of hUC-MSCs**

Using DiR fluorescent dye, prepare a working solution of 1-5 μM and resuspend hUC-MSCs in the dye solution to a density of 1x10^6^ cells/mL. Incubate the hUC-MSCs at 37°C for 20 minutes, then resuspend the cells. Inject the hUC-MSCs labeled with DiR fluorescent dye (experimental group) and PBS (negative control group) into the tail veins of mice, and set different observation times. Use an *In Vivo* Imaging System (IVIS) to track the mice *in vivo*.

**hUC-MSCs tail vein injection**

In the preliminary experiment to explore the injection dose and administration time of hUC-MSCs, we explored it in two steps. In the first stage, based on previous studies, we injected hUC-MSCs into the tail vein once a week for a total of 4 times, and set up three different hUC-MSCs doses of low, medium and high, namely 2×10^5^ cells/mouse, 5×10^5^ cells/mouse and 1×10^6^ cells/mouse. Subsequent analysis showed that 1×10^6^ cells/mouse led to an increased risk of pulmonary embolism-related death in mice, so we chose to inject hUC-MSCs into the tail vein once a week at 2×10^5^ cells/mouse. In the second stage, we injected 2×10^5^ hUC-MSCs per mouse through the tail vein at different ages (9 weeks, 14 weeks, and 21 weeks) of *Col4a5*-deficient mice to evaluate the optimal time for hUC-MSCs to take effect. We evaluated proteinuria and renal tissue pathology and found that earlier treatment with hUC-MSCs can significantly delay the progression of renal fibrosis. Therefore, we chose to inject 2×10^5^ cells/mouse of the passage3 hUC-MSCs into 9-week-old mice, divided into two injections (9 weeks and 11 weeks of age), with an interval of two weeks between the two injections.

**Cell co-culture**

The cell experiment groups consisted of the following categories: the Control Group (CRTL) comprised peripheral blood-induced differentiated podocytes derived from healthy individuals; the Disease Group (AS) included podocytes induced from patients with AS; the Intervention Group (AS+PAN) involved pre-treating AS-hiPS-podocytes with puromycin at 0.5 μg/mL for 24 hours to enhance adhesion; and the Treatment Group (AS+PAN+MSC) entailed co-culturing AS-hiPS-podocytes with hUC-MSCs for 48 hours post a 24-hour PAN stimulation. This process required adjusting the hiPS-podocyte cell concentration to 2 x10^5^ cells/ml, placing them in the lower chamber of a Transwell plate. Concurrently, hUC-MSCs were seeded in the upper chamber at the same concentration, with three replicates per group. Following the 48-hour co-culture period, cells from the lower chamber were removed, the medium discarded, a cell count conducted, and the remaining cells fixed with PFA for subsequent analyses.

**Cytoskeleton staining**

The cell samples were fixed onto a cell slide, permeabilized using 0.5% Triton X-100, washed, and treated with TRITC-labeled phalloidin working solution, followed by a 30-minute incubation at room temperature in the dark. Subsequently, cell nuclei were stained with DAPI solution, the samples were mounted, and TRITC excitation/emission filters (Ex/Em=545/570nm) and DAPI excitation/emission filters (Ex/Em=364/454nm) were selected for imaging. Actin stress fibers were quantified based on the following criteria: Type A: Cell area filled with thick cables, constituting more than 90% of the area. Type B: Presence of at least two thick cables beneath the nucleus, with the remaining area filled with fine cables. Type C: Absence of thick cables, but some cables present. Type D: No visible cables in the central area of the cell. For quantification, four random fields of view were selected. All cells within each field were counted, and the percentage of each cell type within the field was calculated. This method allowed for a detailed assessment of actin stress fiber organization in the cell samples.

**Cell apoptosis analysis**

The Annexin V/7-AAD apoptosis detection assay was conducted following the manufacturer's guidelines. Initially, hiPS-podocytes were seeded in a six-well plate. Subsequent to various treatments, cells were detached using 0.25% trypsin without EDTA, followed by a PBS wash. The cell suspension was then centrifuged at 1500 rpm for five minutes, and the cells were resuspended in 500 μl of staining buffer. Annexin V staining was carried out by adding five μl of Annexin V to the cell suspension, followed by a 15-minute incubation at room temperature in darkness. Subsequently, five μl of 7-AAD reagent was added, and the samples were analyzed using flow cytometry (CytoFLEX, BD, USA) after a 5-minute incubation period.

**Statistical analysis**

For quantitative data, t-tests are used to compare between two groups, while one-way analysis of variance (ANOVA) is used for comparisons among multiple groups. ImageJ 1.45 software is utilized for quantitative analysis of renal pathology staining, where data is represented as the ratio of positive staining area to the total analyzed area. All statistical analyses are conducted using statistical software such as GraphPad Prism 9. A statistically significant difference is considered when P < 0.05.


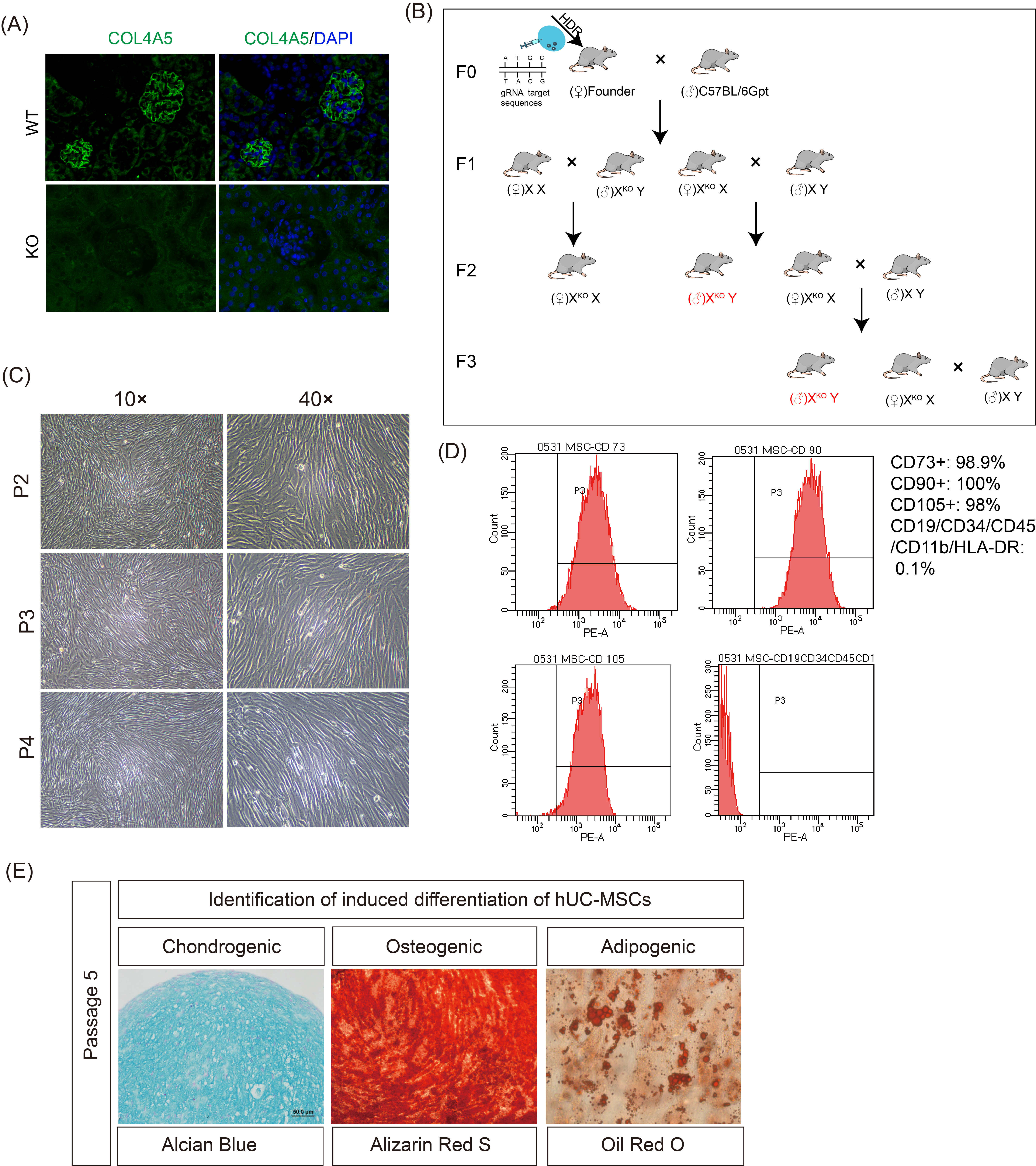


**Figure S1** Validation of *Col4a5* knockout mouse model and characterization of isolated hUC-MSCs. (A) Representative immunofluorescence images demonstrating *Col4a5* deficiency in knockout mouse kidneys. (B) Breeding strategy for generating X-linked Alport syndrome model mice (male Col4a5-/y mice). (C) Appearance of human umbilical cord mesenchymal stem cells at passages 2, 3, and 4. (D) Flow cytometry of hUC-MSCs surface markers CD11b-, CD19-, CD34-, CD45-, HLADR-. CD73+, CD90+, and CD105+. (E) hUC-MSCs induce osteogenic differentiation, adipogenic differentiation and chondrogenic differentiation.


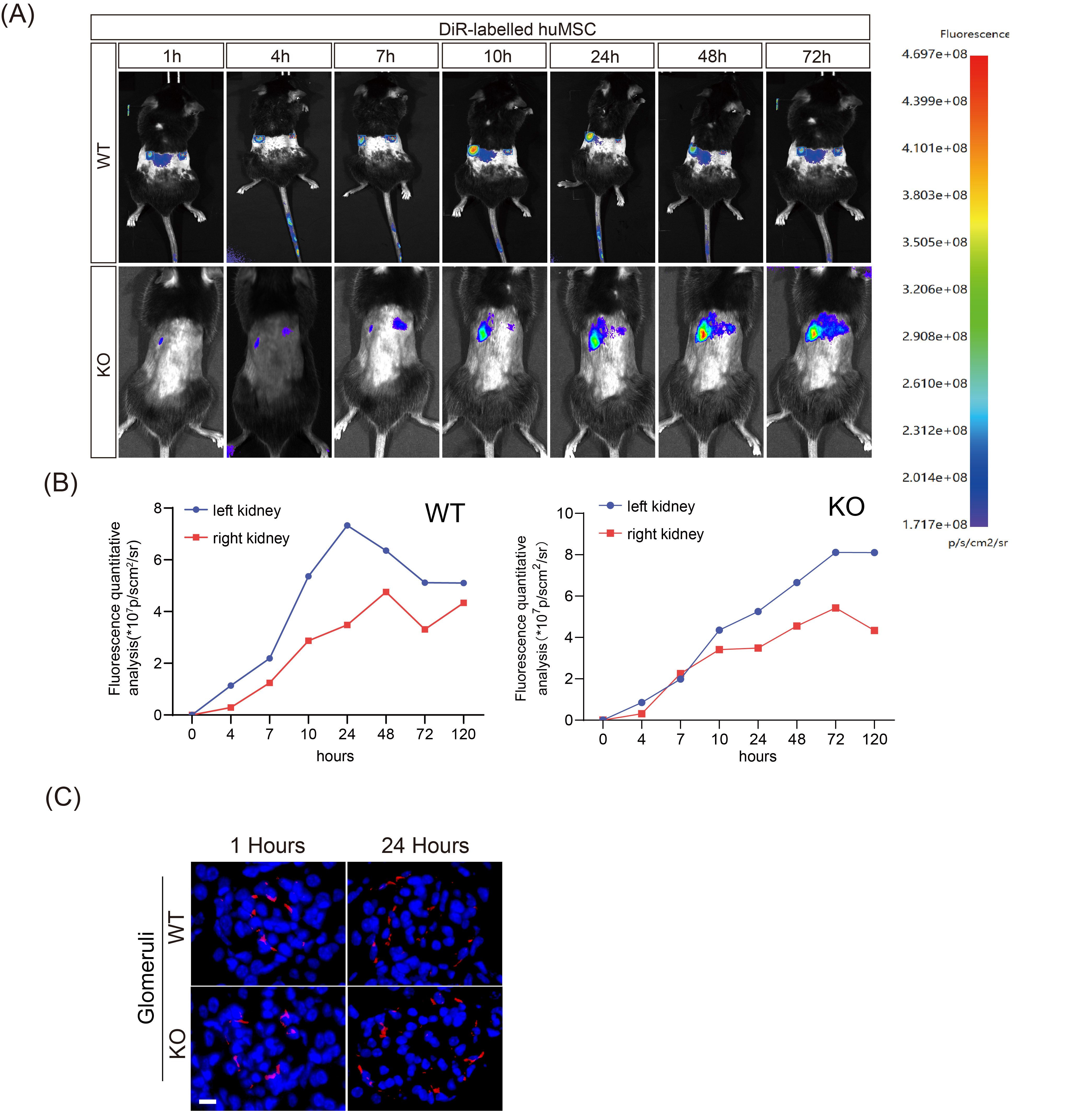


**Figure S2** Distribution of hUC-MSCs in kidneys of WT and *Col4a5* knockout mice. (A-B) Biodistribution analysis of DiR-labeled hUC-MSCs following intravenous administration in WT and KO mice, showing representative in vivo fluorescence imaging (A) and quantitative analysis (B) of kidney-specific fluorescent signals at indicated time points. (C) Fluorescence images of CM-DIL-labeled huMSCs in frozen sections of WT and KO mouse kidneys.


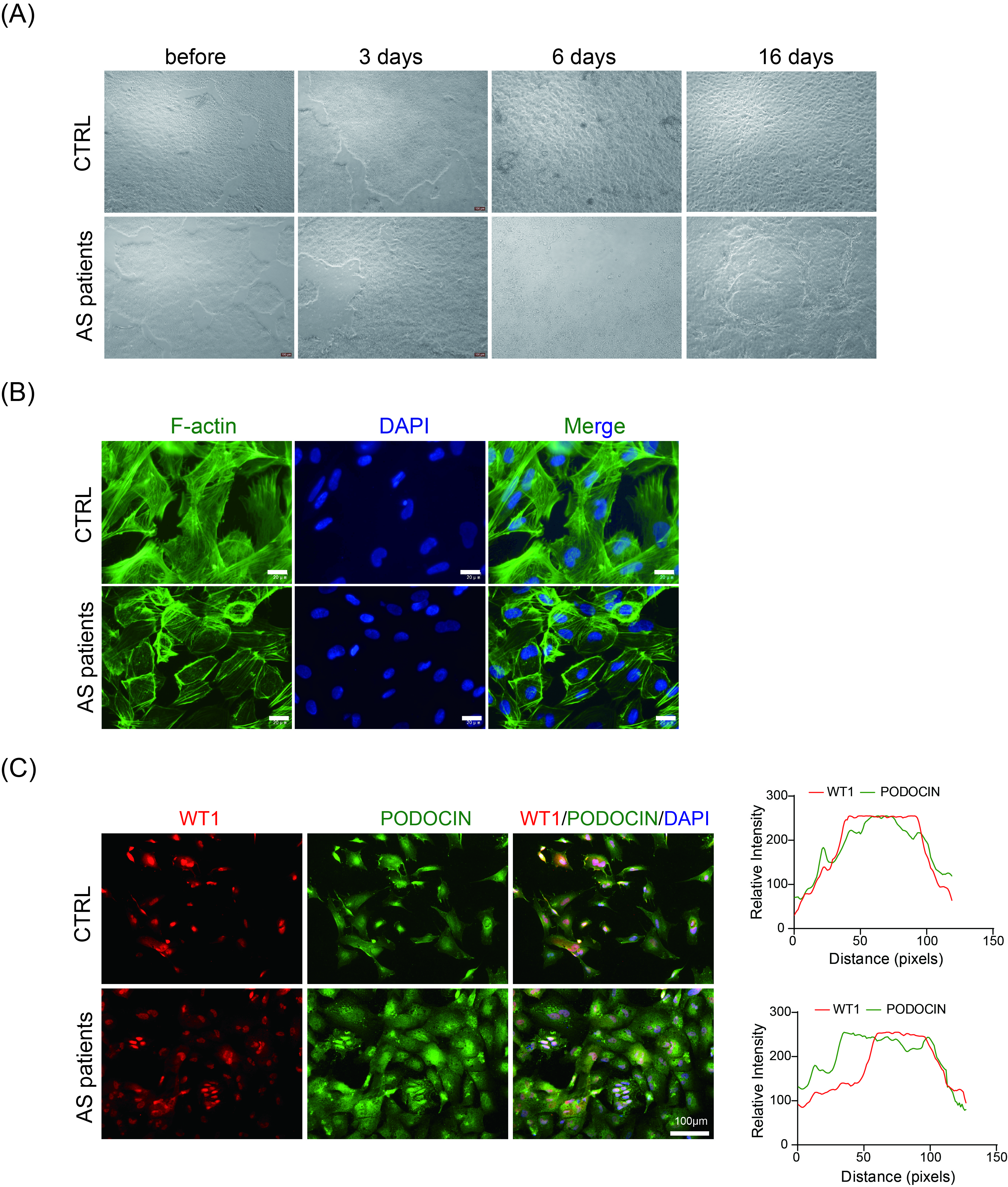


**Figure S3** Identification of renal podocytes differentiated from human induced pluripotent stem cells (hiPSCs). (A) Bright field image of hiPSCs differentiation into renal podocytes. (B) Immunofluorescence of hiPSC-induced differentiation of renal podocyte skeleton. (C) Immunofluorescence of hiPSC-induced differentiation of renal podocytes. Red indicates WT1 positive staining, green indicates Podocin protein positive staining, and DAPI indicates nuclear staining (WT1 and Podocin are both podocyte surface markers). Scale bar 100μm.
